# What factors are associated with maternal undernutrition in eastern zone of Tigray, Ethiopia? Evidence for nutritional well-being of lactating mothers

**DOI:** 10.1186/s12889-020-09313-0

**Published:** 2020-08-08

**Authors:** Semaw Ferede Abera, Eva Johanna Kantelhardt, Afework Mulugeta Bezabih, Mache Tsadik, Judith Lauvai, Gebisa Ejeta, Andreas Wienke, Jan Frank, Veronika Scherbaum

**Affiliations:** 1 Institute of Nutritional Sciences, University of Hohenheim, Stuttgart, Germany; 2 Food Security Center, University of Hohenheim, Stuttgart, Germany; 3 School of Public Health, College of Health Sciences, Mekelle University, Mekelle, Ethiopia; 4 Kilte Awlaelo - Health and Demographic Surveillance Site, College of Health Sciences, Mekelle University, Mekelle, Ethiopia; 5 Institute of Medical Epidemiology, Biostatistics, and Informatics, Faculty of Medicine, Martin-Luther-University, Halle, Germany; 6 Department of Gynaecology, Faculty of Medicine, Martin-Luther-University, Halle, Germany; 7 Department of Agronomy, Purdue University, West Lafayette, IN USA

**Keywords:** Chronic diseases, Adult mortality, Maternal undernutrition, KA-HDSS, Tigray, Ethiopia

## Abstract

**Background:**

Maternal undernutrition is a pervasive health problem among Ethiopian mothers. This study aims at identifying the level of maternal undernutrition and its associated factors in Kilte Awaleo-Health and Demographic Surveillance Site (KA-HDSS), Tigray region, Ethiopia.

**Methods:**

Nutritional status of 2260 lactating mothers was evaluated using the mid-upper-arm circumference (MUAC). Data from the vital events and verbal autopsy databases were linked to the survey and baseline recensus data to investigate the association of adult mortality from chronic causes of death (CoD) on maternal undernutrition. We employed a generalized log-binomial model to estimate the independent effects of the fitted covariates.

**Results:**

The overall prevalence of maternal undernutrition based on MUAC < 23 cm was 38% (95% CI: 36.1, 40.1%). Recent occurrence of household morbidity (adjusted prevalence ratio (adjPR) = 1.49; 95%CI: 1.22, 1.81) was associated with increased risk of maternal undernutrition. In addition, there was a 28% higher risk (adjPR = 1.28; 95%CI: 0.98, 1.67) of maternal undernutrition for those mothers who lived in households with history of adult mortality from chronic diseases. Especially, its association with severe maternal undernutrition was strong (adjusted OR = 3.27; 95%CI: 1.48, 7.22). In contrast, good maternal health-seeking practice (adjPR = 0.86; 95%CI: 0.77, 0.96) and production of diverse food crops (adjPR = 0.72; 95%CI: 0.64, 0.81) were associated with a lower risk of maternal undernutrition. Relative to mothers with low scores of housing and environmental factors index (HAEFI), those with medium and higher scores of HAEFI had 0.81 (adjPR = 0.81; 95%CI: 0.69, 0.95) and 0.82 (adjPR = 0.82; 95%CI: 0.72, 0.95) times lower risk of maternal undernutrition, respectively.

**Conclusions:**

Efforts to ameliorate maternal undernutrition need to consider the influence of the rising epidemiology of adult mortality from chronic diseases. Our data clearly indicate the need for channeling the integrated intervention power of nutrition-sensitive development programs with that of nutrition-specific sectoral services.

## Background

In Ethiopia, recent analyses revealed that most of the millennium development goals were achieved [1], but not the goal for reducing maternal mortality. Although maternal mortality significantly declined, it is still high, especially in rural areas of Ethiopia [1, 2], with maternal nutritional deficiencies being one of the underlying causes [3, 4]. It was estimated that for every maternal death, 1000 women suffer from stunting and/or anemia [5]. These nutritional disorders act synergistically with multiple other factors leading to nutrition-related adverse pregnancy outcomes [5, 6]. Low hemoglobin concentrations, for instance, are associated with postpartum hemorrhage, the leading cause of maternal death [7], due to its attenuating effect on uterine contraction [8, 9]. Apart from its impact on maternal morbidity and mortality, taking into account that the mother and her fetus or newborn are an inseparable dyad, nutritional deficiencies of mothers can cause undernutrition to fetuses and children [5, 10–12]. This in turn can affect their survival and well-being even later during adulthood [13, 14]. Thus, ensuring maternal nutritional well-being plays a critical role for preventing morbidity and mortality of mothers and their children [6].

Maternal undernutrition, however, has remained one of the most serious public health problems to Ethiopian mothers [2, 15–17]. Even though the level of maternal short stature (height less than 145 cm) reported in 2000 was reduced by 41.7% in 2016 in Tigray regional state, there was almost no reduction in the burden of maternal undernutrition [2, 15]. Added to this existing challenge is the paradoxically rising phenomenon of maternal overweight and obesity both nationally and in the regional state, making the efforts of addressing maternal nutritional problems even more complex [2, 18]. Hence, unless maternal undernutrition is alleviated, the health problems resulting from both under- and over-nutrition, which were associated with an increasing burden of non-communicable diseases and intergenerational transmission of poverty [14, 19–21], will be an increasing challenge to public health in the future.

Family planning, antenatal care visit, in-facility delivery, skilled birth attendance and preventing nutritional deficiencies are key inputs to enhancing the survival and well-being of women of reproductive age and their children [22–24]. In contrast, morbidity and mortality of adults from chronic diseases could impose high and regressive costs that could significantly cause household poverty [25–29], thereby influencing maternal nutritional status.

This study was carried out to examine factors associated with undernutrition among lactating mothers using epi-demographic, socio-economic and agricultural datasets from Kilte Awlaelo-Health and Demographic Surveillance Site (KA-HDSS), eastern Tigray, Ethiopia.

## Methods

### Study setting, study period and data sources

Due to poor quality or absence of complete civil registration systems (CVRS), evidence-based health policy formulation and decision-making is challenging in most sub-Saharan Africa [30]. To date, complete and quality CVRS system has not been available in Ethiopia. However, there are other health data sources despite their limitations. In addition to the periodic and routine data sources (censuses, demographic and health survey, routine health information reports), selecting geographically defined populations, and generating longitudinal vital events (birth, migration, and death), causes of death (CoD), and marital status information could also be an alternative systematic approach. Such population-based and ongoing longitudinal health and demographic projects are known as health and demographic surveillance sites (HDSS), and are linked through The International Network for the Demographic Evaluation of Populations and their Health (INDEPTH) network [30, 31]. Pooling data from multiple HDSS, in a given country, may help to narrow the chronic data gaps in low and middle income countries [31], although this approach cannot be a replacement for a fully functional and quality CVRS. In Ethiopia, there are six HDSS projects home-based at their respective local university and networked by the support of Ethiopian Public Health Association (EPHA) and Center for Diseases Control (CDC-ETHIOPIA). Health and demographic data are pooled from these six sites and disseminated for public use on annual basis. KA-HDSS, which is located 835 km north of Addis Ababa in the Eastern Zone of Tigray, was established in 2009 with a total baseline population of 66,438 residents. The current study is based on data extracted from the KA-HDSS longitudinal data, and the nutrition and baseline recensus survey data, which was collected from July to December in 2015. The nutrition survey was conducted as a baseline for the installment of longitudinal nutrition project to the already existing main surveillance project.

### Participants

Our study population included all the 2260 breast-feeding mothers of KA-HDSS, who were at least 18 years old.

### Data collectors and data collection procedure

The data collectors were high school completed permanent employees of the surveillance project (KA-HDSS). They were chosen to ensure the overall sustainability of the project and its collateral contribution to the community. They were rigorously trained, and experienced on the standard data collection tools and procedures, including on how to consent the study participants properly. Before the start of the data collection, the data collectors introduced themselves to the study participants, and gave information on the purpose of the study. The data collection started after consent from the participants was given. Furthermore, the interviewers were residents of the surveillance population, with better knowledge about the culture of their community, thus facilitating the proper implementation of the consent procedures. Face-to-face interviews were conducted together with direct observations and measurements. KA-HDSS data collection supervisors led by the field manager (from the field KA-HDSS office) and academic research members (from the KA-HDSS central home office at Mekelle University, College of Health Sciences) strictly monitored the data collection process.

### Assessment and study variables

Maternal nutritional status was assessed using the mid-upper-arm circumference (MUAC) measurement. The midpoint between the tip of the shoulder and the tip of the elbow of the left arm was measured using a flexible, inelastic MUAC tape. Circumferential measurements were taken after making sure that the tape had a proper tension, not too loose or too tight, around the midpoint and all values were recorded to the nearest 0.1 cm. Lactating mothers with a MUAC below 23 cm were classified as undernourished, and those with less than 21 cm as severely undernourished [32, 33]. Studies have reported that MUAC is a reliably efficient alternative indicator for body mass index for evaluating adult nutritional status, particularly in developing countries where large logistical mobilization is needed to accurately measure the height and weight in population-based surveys [34, 35]. Its positive association with infant breast milk intake, breast milk volume and quality, its measurement simplicity and ability to predict mortality, particularly among the older adults, are additional advantages [35–38].

Detailed lists of agricultural asset ownership, livestock and food crops produced, were counted and converted to monetary terms and quintile classified. The lowest two quintiles were treated as “poor” and the remaining three upper quintiles as “not poor”. Maternal health practice index was constructed from two maternal health service utilization indicators: ever use of modern family planning methods and delivering at health facilities. If the score was 2 (mother used both health services), then it was operationalized as “good”. If the score was < 2 then it was classified as “poor”. A household’s access to media is considered if a given household has access to television, radio or mobile phone. Improved water and sanitation services were computed using the latest WHO/UNICEF Joint Monitoring Program (JMP) ladders for improved water and sanitation sources [39]. The quality of housing materials and cleanliness of cooking fuel were determined by reproducing the approaches used by Adebowale el.al [40]. Principal component analysis was performed on the proportion of nine housing and environmental variables [41]. The nine variables are access to media, electricity, improved water and sanitation services, cleanliness of cooking fuel, availability of kitchen, quality of the housing materials of the floor, wall, and roof. Then, four principal components with eigenvalues ≥1.0 were retained to derive the housing and environmental factors index (HAEFI). The variable agricultural crop diversity was operationalized based on whether the households, with access to farmland, were able to produce at least two food crops. Households with no crop or monocrop production were specified as having no crop diversity. Recent morbidity history was said to be existent if there was report of illness among household member/s 2 weeks before the date of survey. Physician review based on verbal autopsy was employed to ascertain the most likely causes of death (CoD) and all causes were classified using the tenth edition of International Classification of Diseases tool (ICD-10) [42]. Adult deaths from chronic infectious and non-infectious diseases (notably tuberculosis, HIV/AIDS, non-communicable diseases such as diabetes mellitus, hypertension, endocrine disorder and others) were operationally defined as “chronic CoD”. These diseases were classified under the same broad CoD group because these specific CoD may generally be associated with longer duration of illness and need for long-term care. Therefore, relative to all other CoD, chronic CoD might have caused long-term family stress and health care expenditure, and adverse social and economic consequences to the deceased adults (before their death) and their family and household in general. Adult deaths from all acute infectious diseases, external causes like assault, and the undetermined cases were then classified as “all other CoD”. In this study, we hypothesized that maternal undernutrition might be higher for mothers living in households that experienced adult death from chronic CoD, as compared to those living in the households that experienced no adult death or adult death from all other CoD. Investigating maternal nutritional status with such operational classification of the CoD could have empirical relevance to maternal health and nutrition policy since these CoD are contributors to adult health loss in the study community and in Ethiopia in general [43–46].

### Statistical analysis

The factors determining maternal undernutrition were identified using generalized log-binomial model. This statistical procedure was chosen because it can generate estimates of adjusted prevalence ratios (adjPR), appropriate measure of association when the outcome of interest is not epidemiologically rare (> 10%), which is the case in this study [47–50]. In addition, its easiness of interpretation makes prevalence ratio preferable to the odds ratio [50]. Univariable log-binomial analyses were performed to observe the association of each independent variable with maternal undernutrition. In the next step, all the variables with a *P*-value of less than 0.25 in the univariable analysis were taken to the multivariable log-binomial model. Adjusted prevalence ratios and their 95% confidence intervals (95% CI) were reported at *P*-value of less than 0.05. In addition, we also assessed the factors associated with severe maternal undernutrition. In this case, since the prevalence of severe maternal undernutrition was less than 10%, we used multivariable binary logistic regression model to identify the potential associated factors. Similar to the above approach, variable selection was made at *P*-value of less than 0.25 in the univariable binary logistic regression and adjusted odds ratio (AOR) with 95% CI were produced from the multivariable model at *P*-value of less than 0.05. However, to encourage the interpretation of the associations from the perspective of practical (public health) significance, rather than focusing only on statistical significance, we decided to skip the *P*-values and instead report only the measures of association and their corresponding 95% CI. Categorical characteristics of the study participants are presented using frequencies and proportions, while the continuous characteristics are summarized using means with standard deviations (SD) and median with interquartile ranges (IQR), depending on their distribution. All analyses were performed in Stata 15.1 (StataCorp, LLC, College Station, TX).

## Results

### Characteristics of the study participants

Details of socio-demographic and agro-epidemiologic characteristics of the study participants and their households are presented in Table 1. Three-fourths (77.5%) of the households had access to farmland. The median farmland size, for those who had access to farmland, was 2 ha with an IQR of 1–3 ha (data not shown). The median (IQR) altitudinal location of the households, where the undernourished mothers lived in, was slightly higher than their counterparts, 2138 m (2,050–2321) vs. 2102.5 m (2,038 – 2270).

**Table 1:** Description of study participants by nutritional status, KA-HDSS, Tigray, northern Ethiopia (*n* = 2, 260)

Independent variables		Total counts (Column %)	Maternal nutritional status (Count, and row %)	
			Properly nourished	Undernourished
Residence	Rural	2121 (93.8)	1301 (61.3)	820 (38.7)
	Semi-urban	139 (6.2)	99 (71.2)	40 (28.8)
Age in years	Median (IQR^*^)	27 (23–30)	27 (22–31)	28 (23–30)
Age of child in months	Mean (±SD^*^)	10.5 (±6.4)	10.4 (±6.5)	10.8 (±6.3)
Education	No formal education	1372 (62.9)	865 (63.1)	507 (36.9)
	Primary	592 (27.2)	361 (61.0)	231 (39.0)
	Secondary and above	215 (9.9)	125 (58.1)	90 (41.9)
Occupation	Housewife/Farmer	1914 (88.0)	1192 (62.3)	722 (37.7)
	Government employee and others	199 (9.1)	121 (60.8)	78 (39.2)
	Daily laborer/ Unemployed	62 (2.9)	36 (58.1)	26 (41.9)
Asset-based wealth status	Poor	898 (39.7)	573 (63.8)	325 (36.2)
	Not poor	1362 (60.3)	827 (60.7)	535 (39.3)
Household history of adult death	No history adult death	2134 (94.4)	1328 (62.2)	806 (37.8)
	Death from chronic diseases	62 (2.7)	31 (50.0)	31 (50.0)
	Death from all other causes	64 (2.8)	41 (64.1)	23 (35.9)
Maternal health seeking	Poor	1009 (48.8)	601 (59.6)	408 (40.4)
	Good	1059 (51.2)	695 (65.6)	364 (34.4)
HAEFI^*^	Poor	1006 (45.4)	571 (56.8)	435 (43.2)
	Medium	431 (19.5)	288 (66.8)	143 (33.2)
	High	778 (35.1)	514 (66.1)	264 (33.9)
Morbidity in the past 2 weeks	No	2180 (96.5)	1363 (62.5)	817 (37.5)
	Yes	79 (3.5)	36 (45.6)	43 (54.4)
Household size	Mean and SD	6.21 (2.04)	6.25 (2.05)	6.15 (2.03)
Altitude (in meter)	Median and IQR	2110 (2041 – 2300)	2102.5 (2038–2270)	2138 (2050–2321)
Crop diversity	No	1039 (46.0)	590 (62.5)	449 (37.5)
	Yes	1221 (54.0)	810 (45.6)	411 (54.4)

*HAEFI housing and environmental factors index, *IQR* interquartile range, *SD* standard deviation

About one-third of the households (35.1%) had a higher housing and environmental factors index (HAEFI). One hundred and twenty-six (5.5%) of the households had a history of adult deaths and half of these deaths, 62 (49.2%), were from chronic diseases (Table 1). The mean (SD) household size was 6.21 (SD ± 2.04). The median age of the mothers was 27 years IQR of 23 to 30 years. More than three-fifths of the mothers had no formal education and the majority of the women (88%) were either housewives or farmers. The mean maternal MUAC was 23.0 cm with a SD of 1.6. Of the total 2260 mothers, 860 (38%; 95% CI: 36.1, 40.1%) were undernourished (MUAC < 23 to > = 21 cm) and 150 (6.6%%; 95% CI: 5.7, 7.7%) were severely thin (MUAC < 21 cm).

### Household characteristics by agro-economic, infrastructural, housing and environmental factors

Table 2 provides quantile distribution of HAEFI by the component household related characteristics. More than half, 1266 (57.2%), of the households had access to improved water and 499 (22.5%) to sanitation services. Access to improved housing materials, electricity and clean cooking fuel is limited.

**Table 2:** Distribution of housing and environmental factors index (HAEFI) by housing characteristics and access to different public services (*n* = 2215 households) in KA-HDSS, Tigray, Ethiopia

Characteristics		Distribution of HAEFI^*^ (row %)					Total Frequency (Column %)
		Quintile 1	Quintile2	Quintile 3	Quintile 4	Quintile 5	
Floor	Unimproved	30.7	19.0	21.2	16.1	13.0	2026 (91.5)
	Improved	0.0	0.0	1.0	3.2	95.8	189 (8.5)
Wall	Unimproved	34.9	21.6	24.1	15.6	3.8	1781 (80.4)
	Improved	0.0	0.0	0.4	12.7	86.9	434 (19.6)
Roof	Unimproved	37.3	18.3	21.0	13.7	9.7	1221 (55.1)
	Improved	16.8	16.2	17.5	16.7	32.8	994 (44.9)
Access to electricity	No	33.1	20.0	19.0	15.3	12.6	1879 (84.8)
	Yes	0.0	2.4	22.0	13.4	62.2	336 (15.2)
Access to Media	No	5.8	39.9	10.8	26.4	17.1	659 (29.8)
	Yes	37.5	7.8	23.1	10.2	21.3	1556 (70.2)
Kitchen	No	32.9	16.5	18.7	13.2	18.7	614 (27.7)
	Yes	26.2	17.7	19.7	15.7	20.6	1601 (72.3)
Access to water	Unimproved	0.0	0.0	33.8	28.1	38.0	949 (42.8)
	Improved	49.1	30.3	8.7	5.2	6.6	1266 (57.2)
Access to latrine	Unimproved	23.8	20.6	16.5	17.4	21.7	1716 (77.5)
	Improved	42.9	6.2	29.5	7.0	14.4	499 (22.5)
Cooking fuel	unclean/biomass	29.1	18.0	20.1	15.5	17.3	2139 (96.6)
	clean/non-biomass	0.0	0.0	0.0	1.3	98.7	76 (3.4)

*HAEFI Housing and environmental factors index

Generally, improved housing quality, access to electricity and use of non-biomass cooking fuel were observed for mothers in the higher quintile groups of HAEFI. Whereas higher access to improved water and sanitation services was observed for those mothers in the lower HAEFI quintiles (Table 2).

### Factors associated with maternal undernutrition

**Fig. 1:**
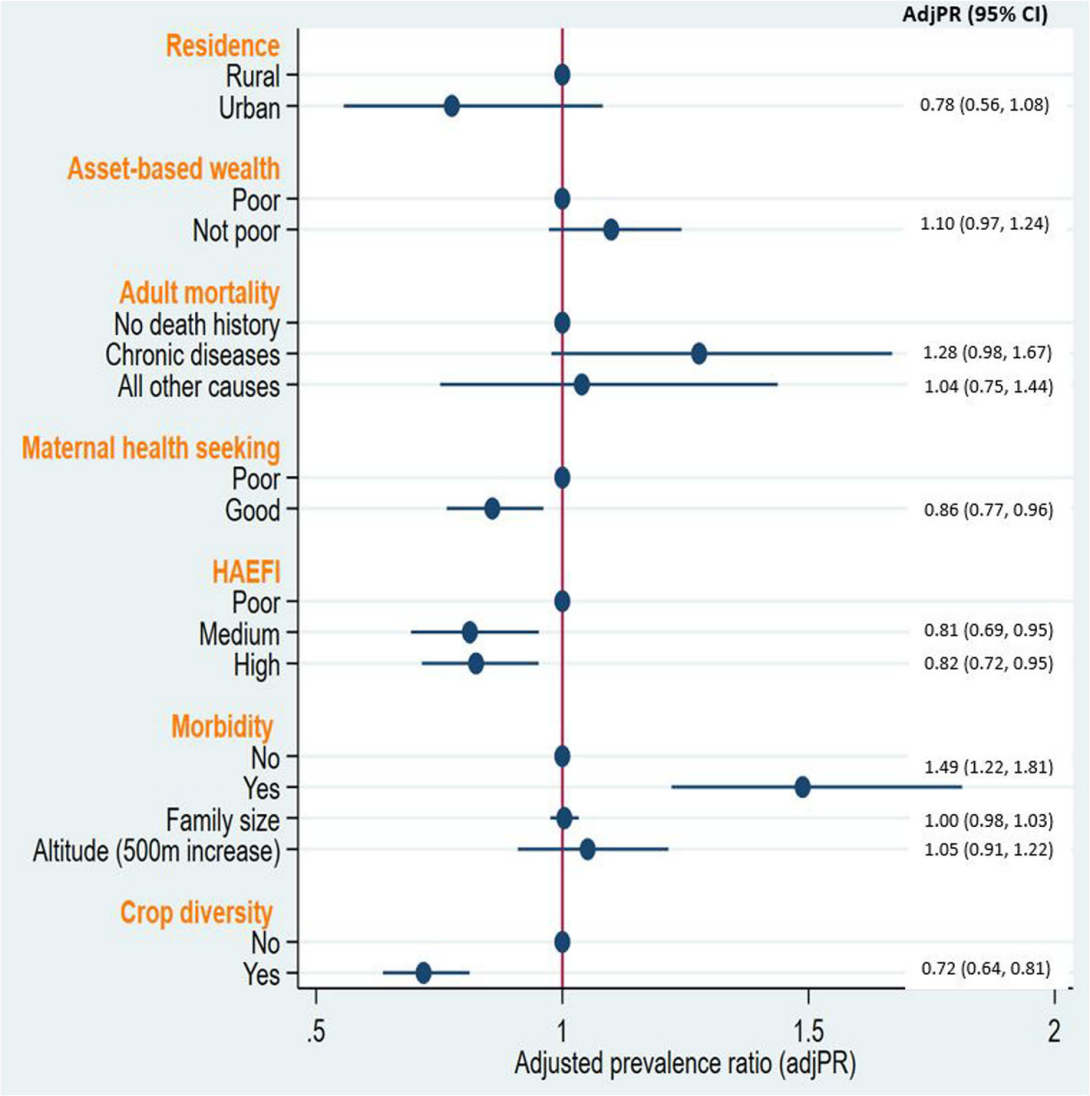
Household factors associated with maternal undernutrition summarized by plots of adjusted PR and 95%CI from multivariable log-binomial model, KA-HDSS, Tigray, Ethiopia. *HAEFI = Housing and environmental factors index, PR = prevalence ratio, CI = confidence interval

Recent history of occurrence of household morbidity (adjPR = 1.49; 95%CI: 1.22, 1.81) was associated with an increased risk of maternal undernutrition. Similarly, household history of adult mortality from chronic diseases (adjPR = 1.28; 95%CI: 0.98, 1.67) was associated with a higher risk of maternal undernutrition with a marginal statistical significance. Compared to those mothers with low HAEFI scores, mothers with medium and higher HAEFI scores had 0.81 (adjPR = 0.81; 95%CI: 0.69, 0.95) and 0.82 (adjPR = 0.85; 95%CI: 0.72, 0.95) times lower risk of undernutrition, respectively (Fig. 1). In addition, good maternal health seeking practice (adjPR = 0.86; 95%CI: 0.7, .96) and diverse food crops production (adjPR = 0.72; 95%CI: 0.64, 0.81) were also associated with lower risk of maternal undernutrition (Fig. 1).

Although higher altitudinal location and rural residence were statistically associated with higher risk of maternal undernutrition in the univariable analysis (Additional file 1), these associations did not hold true when the effects of other fitted variables were adjusted in the multivariable model. Number of household size, mothers’ educational status and occupation were not strongly associated with risk of maternal undernutrition (Fig. 1).

**Fig. 2:**
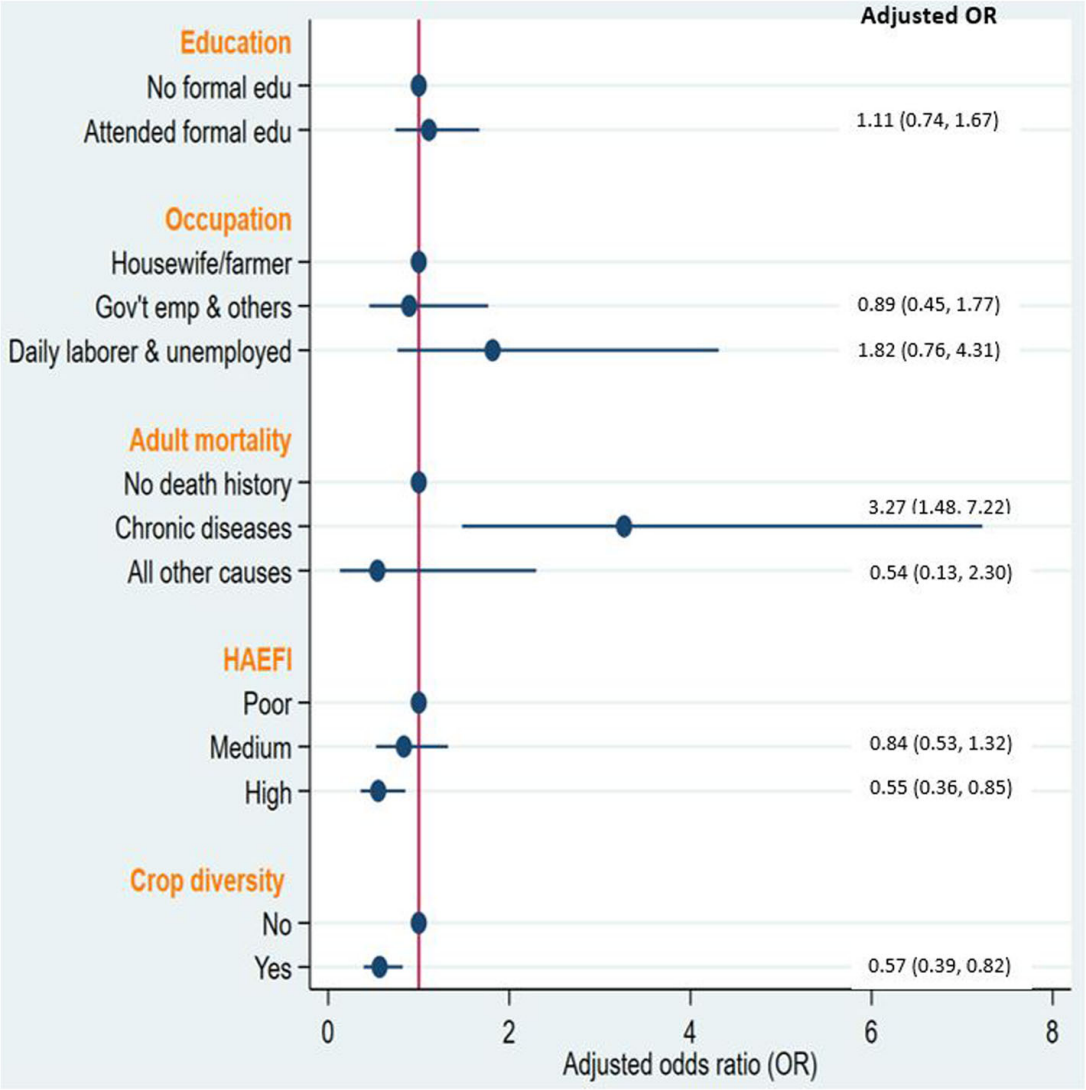
Household factors associated with severe maternal undernutrition summarized by plots of adjusted odds ratio and 95%CI from multivariable binary logistic regression model, KA-HDSS, Tigray, Ethiopia. *HAEFI = Housing and environmental factors index, Gov’t = Government, emp = employees, PR = prevalence ratio, CI = confidence interval

Figure 2 shows that mothers who lived in households with a history of adult mortality from chronic diseases had a 3.27 times (AOR = 3.27; 95%CI: 1.48, 7.22) higher odds of severe maternal undernutrition compared to those who lived in households with no history of adult deaths. The odds of severe maternal undernutrition was 45% (AOR = 0.55; 95%CI: 0.36, 0.85) for mothers who lived in households with a high HAEFI score. Similarly, mothers who lived in households that reported diverse food crops production showed 43% (AOR = 0.57; 95%CI: 0.39, 0.82) lower odds of severe maternal nutrition (Fig. 2). An additional table file shows details of the univariable and multivariable binary logistic regression results (Additional file 2).

## Discussion

This study presents evidence on the level and associated factors of maternal undernutrition in eastern part of Tigray, Ethiopia. The mean MUAC (± SD) of the participants was 23.0 ± 1.6 with an overall prevalence of maternal undernutrition (MUAC< 23 cm) of 38% and severe undernutrition (MUAC< 21 cm) with 6.6%. This study used high quality socio-economic, agro-ecologic and epidemiologic data from community-based surveys implemented in a health and demographic surveillance system platform. Our analysis reveals associations of household histories of adult morbidity and mortality, diverse food crop production, maternal health-seeking practice, housing and environmental factors index with maternal undernutrition.

A study from South Eastern Tigray reported a mean maternal MUAC value of 23.2 cm, which is similar to our finding (MUAC 23.0 cm) [17]. However, the level of severe maternal undernutrition in the cited study area in 2011 was about twice as high as in our study, with a magnitude of 13% vs. 6.6% respectively. Betemariam et al. found even a 24% prevalence of severe maternal undernutrition in Bale Zone of Ethiopia in 2013 [51]. This is nearly four-fold higher than the burden reported in our study, but the study population in that study was from a community that had a marginal agricultural production with cyclic food insecurity, and this may partially explain the observed difference. Similarly, a study based on a large representative survey sample from two Gojjam zones of the Amhara region found 52.9% maternal undernutrition (MUAC< 23 cm), a level much higher than the current finding [52]. The difference in the implementation period of the cited research works, agro-ecologic and cultural variations of the study areas might have contributed to the observed differences. The relatively lower burden of maternal undernutrition in the current study could partly be explained by the fact that the study area is known for being a hot-spot for various health and developmental interventions [53–55]. Nonetheless, the prevalence of maternal undernutrition is still high in our study community.

Morbidity and mortality of household members, particularly from chronic diseases and in developing country settings, could predispose households into a poverty trap [25, 29, 47]. Our data potentially hinted that mothers living in households that experienced adult mortality attributed to chronic diseases had increased risk of undernutrition, compared to those living in households without history of adult mortality. As shown in Fig. 2, the association of households’ experience of chronic diseases attributed adult mortality with severe maternal undernutrition, however, is strong. In our previous work, however, we identified that nutritional insecurity of children aged 6 to 23 months did not vary by households’ history of adult mortality from chronic diseases [56]. This might be explained by the possibility of a buffering effect made by the mothers on the caloric intake of children, which in turn may have resulted in their increased maternal wasting [57]. Therefore, the current finding supports our proposed research hypothesis that the extent of maternal undernutrition, among mothers who lived in households that experienced adult mortality from chronic diseases, may be higher than among those mothers who did not live in such households. In general, chronic diseases are characterized by long duration of illness, with high out-of-pocket medical expenditure. This would more negatively impact nutrition security of poor and uninsured  patients, and affected households could fall into a poverty trap [27, 58–60]. Loss of income, due to lower employment rate of the affected family members or chronic illness and mortality of the affected adults, is also another unavoidable negative consequence imperiling the economic welfare of households [58, 61–63]. Our data suggest that the synergistic negative economic impact of such household-level shocks might ultimately result in undernourishment of mothers, who were living in the households that experienced adult mortality attributed to chronic diseases. Most notably, a study from Ethiopia found a decline in dietary diversity and increase in mean dependency ratio following prime age adult mortality in poor households, regardless of the sex and position of the deceased adult [64]. In a rural setting of South Africa, household food security was affected by adult mortality, particularly by the death of a male wage-earner [65]. A survey conducted 3 years after identification of households affected by adult illness or mortality from HIV/AIDS, in comparison to the non-affected households, were found to have less production of food crops [63]. Furthermore, longitudinal studies linked the effect of adult mortality on the well-being of older household members; accordingly, a sharp drop in body mass index in the short term (possibly related grief and depression) and an increased probability of acute illnesses (explained by increased working hours in the field) in the long-term, were reported [62, 66]. We assume that this effect could plausibly be more intense for the lactating mothers, considering the cultural context in the study community that the mothers are the pillars and main caregivers to family members in addition to bearing the different laborious out-door responsibilities, especially if the husband is deceased. While this assumption needs to be tested in independent studies, we may conclude that lactating mothers living in households that experienced adult death due to chronic diseases are vulnerable to undernutrition. Thus, we strongly recommend that those lactating mothers, and their children, need targeted nutritional screening with a subsequent intervention for those who are already undernourished or found to be at a high risk of undernutrition.

Mortality of adult household member, especially if the deceased one is of prime-age, is associated with adverse income and assets shocks. Illness or mortality of male household head has been shown to lead to a lower crop production, severe impacts on farm production and livestock assets [63, 67]. A study conducted in a district proximal to our study community reported that lower farmland size and not cultivating maize were associated with severe undernutrition (MUAC< 21 cm) of lactating mothers [17]. Our observation, of a 28% lower risk of being undernourished for lactating mothers living in households which produced diversified food crops accords with these research findings. Another study reported a 27% reduction in total cultivated farmland following death of male head or spouse [67]. In our study, the mean household farmland size was significantly lower among the undernourished mothers than those who were not (mean difference was 0.28 ha; T_calc_ = 4.6189, *p* < 0.001). Additionally, HIV/AIDS-related adult mortality is associated with poor agricultural and resource management, such as watershed and soil conservation, diminished care to household family members, mostly felt by women, change in crop mix and lower capacity to ensure food security [67, 68]. This may impede the capacity to cultivate nutritious and diverse food crops and result in undernourishment of the lactating mothers.

We could show that there was a 14% lower risk of maternal undernutrition given the condition that the lactating mother had achieved a good maternal health seeking practice. The nutritional benefit of utilizing maternal health service has also been demonstrated by other studies [17, 51, 69]. Access to such infrastructural services, especially in rural areas, are clear determinants of maternal and child health [70, 71]. Our study revealed that better housing and environmental factor index scores are negatively associated with maternal undernutrition. This composite index was computed based on a number of relevant public health measures such as quality of housing materials, media access and cleanliness of cooking fuel, availability of environmental health services like access to improved water and latrine services. Media exposure increases maternal awareness, and so could positively influence health service utilization, such as antenatal care service [72], which in turn may increase the mothers’ nutritional knowledge. Poor access to water and sanitation services were shown to exert the burden of water collection for the mother and reduces time for mother-child interaction, endangering nutritional well-being of both the mothers and the children [71, 73]. Given that the lactation period is already characterized by higher energy and nutrient requirements of the mother [74–76], the workload to fetch water may further increase energy requirements and enhance the risk of undernutrition. Our data indicate that the levels of moderate and severe maternal undernutrition were higher for mothers who did not have access to improved water relative to those who had access to improved water (X^2^_calc_ = 20.8, *p* < 0.001), which is in line with earlier research showing the protective effect of improved water and handwashing on maternal undernutrition [77, 78].

Our study poses some limitations. Firstly, the surveillance site, source of the study data, was established not only with the aim of generating scientific health and demographic information, but also to serve as a platform for the implementation of various researches. Due to its proximity to the regional city and thus to Mekelle University, the study area has also been one of the sites for the community-based training program (CBTP) of the College of Health Sciences of the University. In this practical attachment program teams of health sciences and medical students are routinely involved in assessing the general health status of the community, designing, and implementing interventions for sets of identified and prioritized public health problems. This has been the tradition for many years to equip graduating students with various medical and public health skills. Because of these exposures, we may assume that the frequently exposed part of the study site’s population could have a better health literacy level and healthy behavior compared to other communities. If this assumption holds true, the strength of the association of adult mortality from chronic CoD with maternal undernutrition might have been attenuated. However, we do not have the data to evaluate if this bias is induced to the estimates. Secondly, the association of maternal undernutrition with adult mortality attributed to chronic CoD is only at aggregate level and does not give detailed information of each of the chronic causes of adult death. Thirdly, the degree of maternal undernutrition may vary by the length of duration since the occurrence of adult death and the current analyses did not take into account this probable source of variation. Notwithstanding these limitations, our study is based on extensive and high-quality data, and its findings could be relevant to maternal nutrition and to the public health actors in general.

## Conclusion

The level of undernutrition among the lactating mothers of the current study community was high. The factors identified in this study have empirical relevance to public health and nutrition policy. We observed that household morbidity history and adult mortality from chronic diseases were associated with increased risk of maternal undernutrition. On the other hand, good maternal health seeking practice, diverse food crops production, and higher scores of housing and environmental factors index were associated with better maternal undernutrition.

The current findings suggest the need for designing and integrating action-oriented nutrition-sensitive development programs (agriculture and economic sectors) to nutrition-specific interventions (health and nutrition interventions) and channeling such synergistic intervention to predominantly rural households of Ethiopia. This could sustainably improve nutritional well-being of mothers and children. A new and important aspect to consider is that lactating mothers living in households that experienced adult death from chronic diseases need targeted nutritional screening and intervention.

## Supplementary information

**Additional file 1:Supplementary Table 1.** Factors associated with maternal undernutrition, identified by multivariable log-binomial analysis in KA-HDSS, Tigray, Ethiopia Ethiopia.

**Additional file 2:Supplementary Table 2.** Factors associated with severe maternal undernutrition, identified by multivariable binary logistic regression analysis in KA-HDSS, Tigray, Ethiopia.

## Data Availability

The underlying data were extracted from both the Nutrition Survey and an ongoing surveillance site, KA-HDSS, and both are solely the property of KA-HDSS. The datasets analyzed during the current study are not publicly available because the data contain some sensitive information, such as causes of death for the deceased individuals along with GPS identified household locations. But, the data can be accessed from the corresponding author on reasonable request.

## Abbreviations

adjPR: Adjusted Prevalence Ratio
AOR: Adjusted Odds Ratio
CBTP: Community-Based Training Program
CDC: Centers for Disease Control and Prevention
CI: Confidence Interval
CoD: Causes of Death
EPHA: Ethiopian Public Health Association
ERC: Ethics Review Committee
FSC: Food Security Centre
HAEFI: Housing and Environmental Factors Index
HIV/AIDS: Human Immunodeficiency Virus/ Acquired Immunodeficiency Syndrome
HRERC: Health Research Ethics Review Committee
ICD: International Classification of Diseases
IQR: Interquartile range
JMP: Joint Monitoring Program
KA-HDSS: Kilte Awaleo-Health and Demographic Surveillance Site
MUAC: Mid-Upper-Arm Circumference
SD: Standard Deviation
UNICEF: The United Nations Children’s Fund
WHO: World Health Organization
