# Supplementary material for: What factors are associated with maternal undernutrition in eastern zone of Tigray, Ethiopia? Evidence for nutritional well-being of lactating mothers
Source: BMC Public Health. 2020 Aug 8;20:1214. doi: 10.1186/s12889-020-09313-0 (PMC7414570; doi:10.1186/s12889-020-09313-0)
Supplement: Supplementary file 1 — Additional file 1:Supplementary Table 1. Factors associated with maternal undernutrition, identified by multivariable log-binomial analysis in KA-HDSS, Tigray, Ethiopia Ethiopia. [file 12889_2020_9313_MOESM1_ESM.docx]

**Supplementary Table 1.** Factors associated with maternal undernutrition, identified by multivariable log-binomial analysis in KA-HDSS, Tigray, Ethiopia

| **Independent variables** | | **Maternal**  **undernutrition** | |
| --- | --- | --- | --- |
|  |  | **Crude PR**  **(95% CI)** | **Adjusted PR**  **(95% CI)** |
| Residence | Rural | 1.00 | 1.00 |
|  | Semi-urban | 0.74 (0.57, 0.97) | 0.78 (0.56, 1.08) |
| Age (5 year increase) |  | 0.98 (0.94 , 1.03) |  |
| Education | No formal education | 1.00 |  |
|  | Primary | 1.06 (0.93, 1.19) |  |
|  | Secondary and above | 1.13 (0.95, 1.35) |  |
| Occupation | Housewife/Farmer | 1.00 |  |
|  | Government employee and others | 1.04 (0.87, 1.25) |  |
|  | Daily laborer/ Unemployed | 1.11 (0.82, 1.50) |  |
| Asset-based wealth status | Poor | 1.00 | 1.00 |
|  | Not poor | 1.08 (0.97, 1.21) | 1.10 (0.97, 1.24) |
| Household history of adult death | No history adult death | 1.00 | 1.00 |
|  | Death from chronic diseases | 1.32 (1.03, 1.71) | 1.28 (0.98, 1.67) |
|  | Death from all other causes | 0.95 (0.68, 1.33) | 1.04 (0.75, 1.44) |
| Maternal health seeking | Poor | 1.00 | 1.00 |
|  | Good | 0.85(0.76, 0.95) | 0.86 (0.77, 0.96) |
| Housing and environmental factors index | Poor | 1.00 | 1.00 |
|  | Medium | 0.77 (0.66, 0.89) | 0.81 (0.69, 0.95) |
|  | High | 0.78 (0.69, 0.88) | 0.82 (0.72, 0.95) |
| Morbidity in the past 2 weeks | No | 1.00 | 1.00 |
|  | Yes | 1.45 (1.18, 1.79) | 1.49 (1.22, 1.81) |
| Household size |  | 0.98 (0.96, 1.01) | 1.00 (0.98, 1.03) |
| Altitude (500 meter increase) |  | 1.13 (1.00, 1.28) | 1.05 (0.91, 1.22) |
| Crop diversity | No | 1.00 | 1.00 |
|  | Yes | 0.78 (0.70, 0.87) | 0.72 (0.64, 0.81) |
