# Supplementary material for: What factors are associated with maternal undernutrition in eastern zone of Tigray, Ethiopia? Evidence for nutritional well-being of lactating mothers
Source: BMC Public Health. 2020 Aug 8;20:1214. doi: 10.1186/s12889-020-09313-0 (PMC7414570; doi:10.1186/s12889-020-09313-0)
Supplement: Supplementary file 2 — Additional file 2:Supplementary Table 2. Factors associated with severe maternal undernutrition, identified by multivariable binary logistic regression analysis in KA-HDSS, Tigray, Ethiopia. [file 12889_2020_9313_MOESM2_ESM.docx]

**Supplementary Table 2.** Factors associated with severe maternal undernutrition, identified by multivariable binary logistic regression analysis in KA-HDSS, Tigray, Ethiopia

| **Independent variables** | | **Severe maternal**  **undernutrition** | |
| --- | --- | --- | --- |
|  |  | **Crude OR**  **(95% CI)** | **Adjusted OR**  **(95% CI)** |
| Residence | Rural | 1.00 |  |
|  | Semi-urban | 0.64 (0.29, 1.41) |  |
| Age (5 year increase) |  | 0.98 (0.85, 1.14) |  |
| Education | No formal education | 1.00 | 1.00 |
|  | Attended formal education | 1.24 (0.87, 1.77) | 1.11 (0.74, 1.67) |
| Occupation | Housewife/Farmer | 1.00 | 1.00 |
|  | Government employee  and others | 0.98 (0.53, 1.83) | 0.89 (0.45, 1.77) |
|  | Daily laborer/ Unemployed | 1.93 (0.84, 4.43) | 1.82 (0.76, 4.31) |
| Asset-based wealth status | Poor | 1.00 |  |
|  | Not poor | 1.10 (0.78, 1.55) |  |
| Household history of adult death | No history adult death | 1.00 | 1.00 |
|  | Death from chronic diseases | 2.81 (1.31, 6.03) | 3.27 (1.48, 7.22) |
|  | Death from all other causes | 0.95 (0.33, 2.68) | 0.54 (0.13, 2.30) |
| Maternal health seeking | Poor | 1.00 |  |
|  | Good | 0.96 (0.67, 1.38) |  |
| HAEFI | Poor | 1.00 | 1.00 |
|  | Medium | 0.80 (0.51, 1.26) | 0.84 (0.53, 1.32) |
|  | High | 0.63 (0.42, 0.94) | 0.55 (0.36, 0.85) |
| Morbidity in the past 2 weeks | No | 1.00 |  |
|  | Yes | 1.58 (0.65, 3.81) |  |
| Household size |  | 0.97 (0.89, 1.05) |  |
| Altitude (500 meter increase) |  | 0.96 (0.63, 1.47) |  |
| Crop diversity | No | 1.00 | 1.00 |
|  | Yes | 0.56 (0.40, 0.78) | 0.57 (0.39, 0.82) |
